# Dynamic Changes in Serum 25-Hydroxyvitamin D during Pregnancy and Lack of Effect on Thyroid Parameters

**DOI:** 10.1371/journal.pone.0090161

**Published:** 2014-03-07

**Authors:** Yuhang Zhao, Wei Miao, Chenyang Li, Xiaohui Yu, Zhongyan Shan, Haixia Guan, Weiping Teng

**Affiliations:** 1 Department of Endocrinology and Metabolism, Institute of Endocrinology, Liaoning Provincial Key Laboratory of Endocrine Diseases, The First Affiliated Hospital of China Medical University, Shenyang, Liaoning Province, People's Republic of China; 2 Department of Gynecology and Obstetrics, Shenyang Women's and Children's Hospital, Shenyang, Liaoning Province, People's Republic of China; Gentofte University Hospital, Denmark

## Abstract

**Background & Aims:**

To explore vitamin D status and its dynamic changes during pregnancy in women living in Northeast China. The association between 25-hydroxyvitamin D and serum calcium, phosphate and parathyroid hormone was studied. Because vitamin D deficiency or thyroid dysfunction/autoimmunity during pregnancy may lead to similar adverse events, the relationship between 25-hydroxyvitamin D and thyroid parameters was investigated.

**Methods:**

Serum samples of 50 women (aged 22 to 36 years) were selected retrospectively. The samples were collected at gestational 8 weeks ±3 days, 20 weeks ±3 days and 32 weeks ±3 days for measurement of 25-hydroxyvitamin D, calcium, phosphate, parathyroid hormone, and thyroid parameters.

**Results:**

The median 25-hydroxyvitamin D levels were 28.29, 39.23 and 40.03 nmol/L, respectively, from the first to the third trimester. The 25-hydroxyvitamin D concentration during the first trimester was significantly lower than the next two trimesters (p<0.01) and was unchanged between the second and the third trimester. Of these women, 96%, 78% and 76% showed 25-hydroxyvitamin D ≤50 nmol/L during each trimester. Season was associated with 25-hydroxyvitamin D during each trimester (p<0.05), and a significant association was found between calcium and 25-hydroxyvitamin D during the first and the second trimesters. Only triiodothyronine was associated with 25-hydroxyvitamin D in the first trimester (p = 0.024), but statistical significance was only a trend (p = 0.063) after excluding abnormal values. No association was observed between 25-hydroxyvitamin D and phosphate, parathyroid hormone, and other thyroid parameters.

**Conclusions:**

Vitamin D deficiency during pregnancy was prevalent in women from Northeast China who did not use supplementation. No significant relationships were observed between 25-hydroxyvitamin D and thyroid parameters during pregnancy.

## Introduction

The important role of vitamin D in the regulation of calcium homeostasis and maintenance of bone health has long been recognized. Sources of vitamin D include cutaneous synthesis via exposure to sunlight, which accounts for about 95%, and dietary intake for the remaining 5% [1]. Generally, the serum 25-hydroxyvitamin D (25(OH)D) level is the best indicator of overall vitamin D status, because this measurement reflects total vitamin D from sunlight exposure and diet, as well as the conversion of vitamin D from adipose stores in the liver [2], [3]. Several factors influence serum 25(OH)D concentrations including latitude [4], season [5], dietary habits [6], race/ethnicity [7], [8], cultural and religious factors [9]–[11], smoking and drinking [12], supplementation [13], sunscreen use [14], education [12], and body mass index (BMI) [15]. To date, vitamin D deficiency in pregnancy is still a public health issue [16], and increasing evidence shows that vitamin D deficiency/insufficiency leads to a series of adverse outcomes for pregnant women and their offspring.

In addition to the classical roles of vitamin D, it also has been recognized to be a crucial immunomodulator. The biological effects of vitamin D are carried out by its binding to vitamin D receptors (VDRs), which are found in many immunocytes and tissues, such as T lymphocytes, macrophages, dendritic cells, monocytes [17] and thyrocytes [18]. It has been reported that vitamin D deficiency and/or VDRs gene polymorphism could influence cytokine expression, differentiation, and maturation, as well as apoptosis of immunocytes [19]–[21]. An *in vitro* study found that calcitriol (1,25(OH)_2_D) potently attenuated the thyroid-stimulating hormone (TSH)-stimulated production of the intracellular signaling molecule cyclic adenosine monophosphate, iodide uptake, and growth of thyrocytes [18]. Furthermore, clinical trials have demonstrated that vitamin D deficiency and/or VDRs gene polymorphism were associated with many kinds of autoimmune disorders, including type 1 diabetes mellitus [22], systemic lupus erythematosus [23], multiple sclerosis [24], rheumatic arthritis [25], and thyroid autoimmunity with or without overt or subclinical hypothyroidism [26]–[28].

Coincidentally, both vitamin D deficiency and thyroid dysfunction/autoimmunity during pregnancy can lead to some of the same adverse events, such as preeclampsia [29], [30], gestational hypertension [29], [31], gestational diabetes mellitus [32], [33], premature delivery [31], [34], low birth weight [30], and impaired neurodevelopment of offspring [36], [37]. It is not known whether these two disorders share the same mechanisms. Due to a lack of data from North China, the aims of the present study were to observe changes in serum 25(OH)D levels in pregnant women who declared that they did not use vitamin D or calcium supplementation before or during pregnancy, and to examine the relationship between 25(OH)D concentrations and thyroid parameters.

## Subjects and Methods

### Subjects

The blood samples of 50 Chinese patients aged 22–36 years who resided in Northeast China (latitude 41°N) were included in this study. The subjects were selected retrospectively from 342 women who attended routine obstetric examinations at The Fifth People's Hospital of Shenyang City from May 2005 to March 2007. The patients' blood samples were withdrawn from the sample bank after assessment. The inclusion criteria were: (1) blood samples collected from the 1^st^ trimester to the 3^rd^ trimester at gestational 8 weeks ±3 days, 20 weeks ±3 days and 32 weeks ±3 days, respectively; (2) no history of serious illness; (3) subjects did not use vitamin D or calcium supplementation before or during pregnancy; (4) no history of medications affecting thyroid function and metabolism of vitamin D and calcium before or during pregnancy; and (5) no pregnancy-related complications such as hyperemesis gravidarum, trophoblast cell disease or preeclampsia. The study was approved by the Institutional Review Board of China Medical University. The detailed protocol was explained to the patients and written informed consent was obtained from every subject.

### Measurements

Fasting blood was drawn. Serum was separated and maintained at −80°C until analysis. Serum 25(OH)D and parathyroid hormone (PTH) were measured by electrochemiluminescence immunoassay (ECLA) using Roche commercial kits (cobas e 601, Roche, Germany). Serum calcium (Ca) and phosphate (PHOS2) were assayed by colorimetry using Roche commercial kits (cobas c 501, Roche, Germany). Serum thyroid stimulating hormone (TSH), free triiodothyronine (FT_3_), free thyroxine (FT_4_), thyroid peroxidase antibody (TPOAb) and thyroglobulin antibody (TgAb) were analyzed by solid-phase chemiluminescence enzyme immunoassay using IMMULITE 1000 kits (Siemens Healthcare Diagnostics, Tarrytown, NY, USA). Both intra- and inter-assay coefficients of variation of all of the commercial kits were <10%.

### Definitions

For the purpose of the present study, vitamin D status was defined as vitamin D deficiency (25(OH)D ≤50 nmol/L), insufficiency (50 nmol/L<25(OH)D<75 nmol/L) and sufficiency (25(OH)D≥75 nmol/L). These cutoff-points were based on *Evaluation, treatment, and prevention of vitamin D deficiency: an Endocrine Society clinical practice guideline* [38]. We defined months from July to October as summer (S), and November to May as winter (W). According to different months of samples collection, we classified each trimester into S and W subgroups, and S and W also were seen as season in the multiple linear regression analysis.

### Statistical analysis

All data were entered into a database and statistical analysis was carried out with SPSS software (version 16.0; Chicago, USA). The results are presented as the medians with the 25^th^ and the 75^th^ percentiles in parentheses unless indicated otherwise. The comparison of normal distributed data among each trimester was carried out with ANOVA for repeated measures and that of non-normal distributed data (including serum PHOS2) was carried out using the K-related samples test. The comparison between S and W subgroups was carried out using a *t* test (two-tailed). The rate comparison was performed using Fisher's exact test. The relationship between 25(OH)D and the other parameters was carried out using a stepwise, multiple linear regression model. Results were considered significant when p<0.05.

## Results

### Vitamin D deficiency during pregnancy

The median age of the 50 women was 28 years with the 25^th^ and the 75^th^ percentiles of 26 and 32 years, respectively. Parameters of the study subjects are summarized in Table 1. The prevalence of vitamin D deficiency was surprisingly high from the 1^st^ trimester to the last trimester, at 96%, 78% and 76%, respectively. The rate of vitamin D deficiency in the 1^st^ trimester was significantly higher than in the remaining trimesters (p<0.01). Furthermore, few subjects (n = 0, n = 1, n = 3, respectively) were able to achieve vitamin D sufficiency during any trimester (Table 2).

**Table 1:** Study parameters.

Parameters	The 1^st^ trimester	The 2^nd^ trimester	The 3^rd^ trimester
25(OH)D (nmol/L) Total	28.29 (23.46, 35.49)	39.23 (28.38, 49.51)	40.03 (28.69, 49.70)
S Subgroup	30.82 (23.98, 42.95)	42.34 (35.32, 54.06)	48.15 (29.50, 66.23)
W Subgroup	26.38 (22.43, 32.73)	30.64 (24.37, 39.39)	34.65 (27.25, 44.14)
Ca (mmol/L)	2.22 (2.15, 2.27)	2.12 (2.07, 2.20)	2.10 (2.02, 2.16)
PHOS2 (mmol/L)*	1.21 (1.07, 1.37)	1.18 (1.14, 1.33)	1.29 (1.14, 1.41)
PTH (pg/mL)	10.72 (8.59, 14.70)	11.43 (8.19, 15.54)	11.96 (9.25, 18.03)
TSH (mIU/L)	1.155 (0.695, 2.498)	1.045 (0.780, 1.550)	1.020 (0.765, 1.730)
FT_3_ (pmol/L)	4.50 (4.00, 5.20)	5.40 (4.80, 6.33)	5.30 (4.73, 6.05)
FT_4_ (pmol/L)	17.55 (16.43, 19.60)	14.50 (13.05, 15.30)	13.50 (12.20, 14.93)
TPOAb (IU/mL)#*	113.03 (42.38∼183.68)	68.52 (21.26∼115.79)	48.16 (10.27∼86.06)
TgAb (IU/mL)#*	79.05 (18.10∼140.00)	44.43 (18.91∼69.95)	46.60 (4.24∼88.95)

Abbreviations: 25(OH)D, 25-hydroxyvitamin D; S, summer; W, winter; Ca, calcium; PHOS2, phosphate; PTH, parathyroid hormone; TSH, thyroid stimulating hormone; FT_3_, free triiodothyronine; FT_4_, free thyroxine; TPOAb, thyroid peroxidase antibody; TgAb, thyroglobulin antibody.

Data are given as medians with the 25^th^ and the 75^th^ percentiles in parentheses unless indicated otherwise; #, data are presented as means with 95% confidence intervals in parentheses;

*, data do not conform to a normal distribution.

**Table 2:** Changes of vitamin D status during pregnancy.

25(OH)D status (nmol/L)	The 1^st^ trimester n (%)	The 2^nd^ trimester n (%)	The 3^rd^ trimester n (%)
25(OH)D≤50	48 (96)*	39 (78)	
50<25(OH)D<75	2 (4)	10 (20)	9 (18)
25(OH)D≥75	0 (0)	1 (2)	3 (6)

Abbreviation: 25(OH)D, 25-hydroxyvitamin D.

25(OH)D≤50, vitamin D deficiency; 50<25(OH)D<75, vitamin D insufficiency; 25(OH)D≥75, vitamin D sufficiency;

*, p<0.01 compared to the 2^nd^ and the 3^rd^ trimester.

### Changes in 25(OH)D, Ca, PHOS2 and PTH during pregnancy

**Figure 1:**
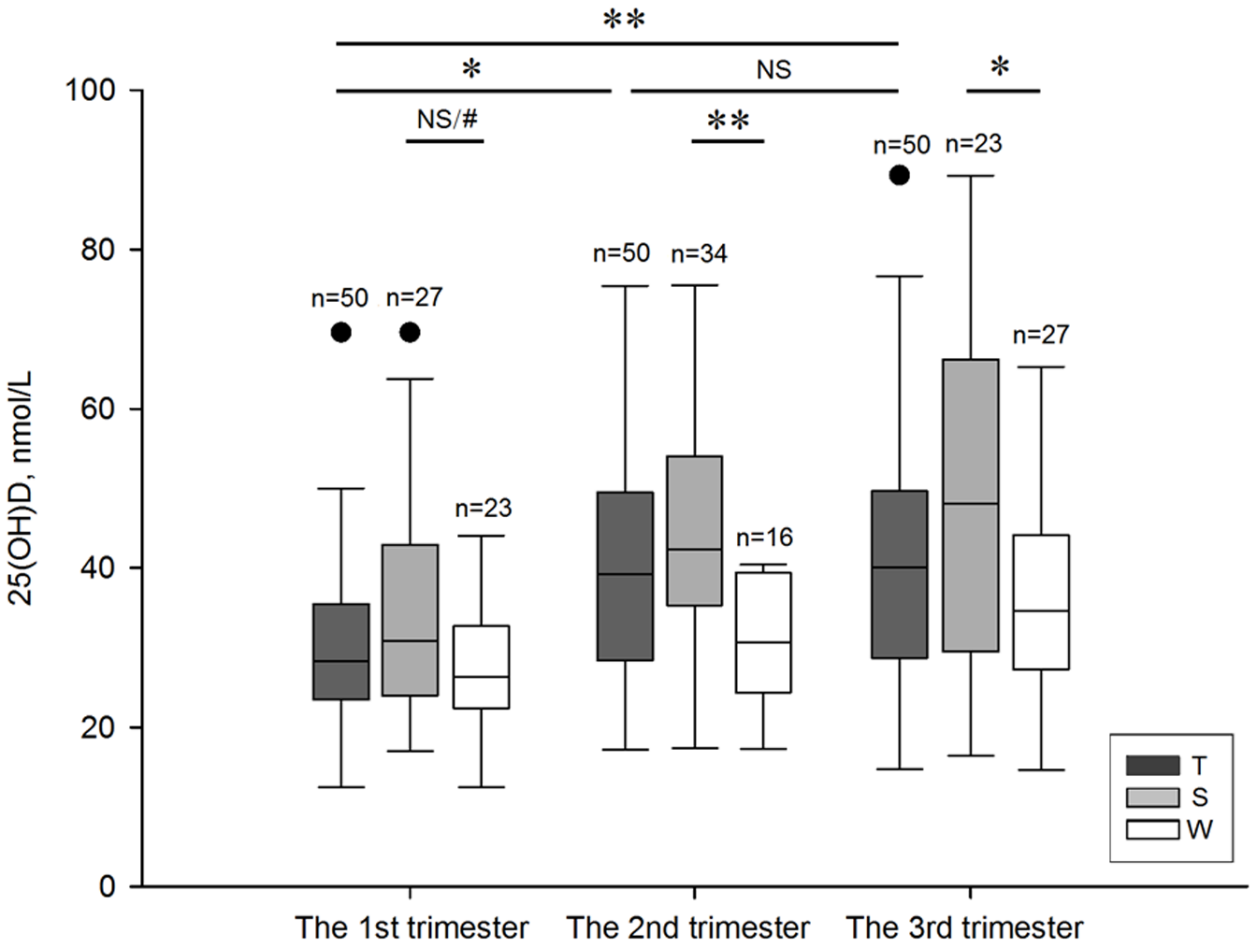
Changes in 25(OH)D during pregnancy. Data are presented as boxplots. 25(OH)D, 25-hydroxyvitamin D; T, total; S, summer; W, winter; NS, not significant; •, outliers; *, p = 0.005; **, p = 0.000; #, p = 0.031 when the significance level one-tailed.

**Figure 2:**
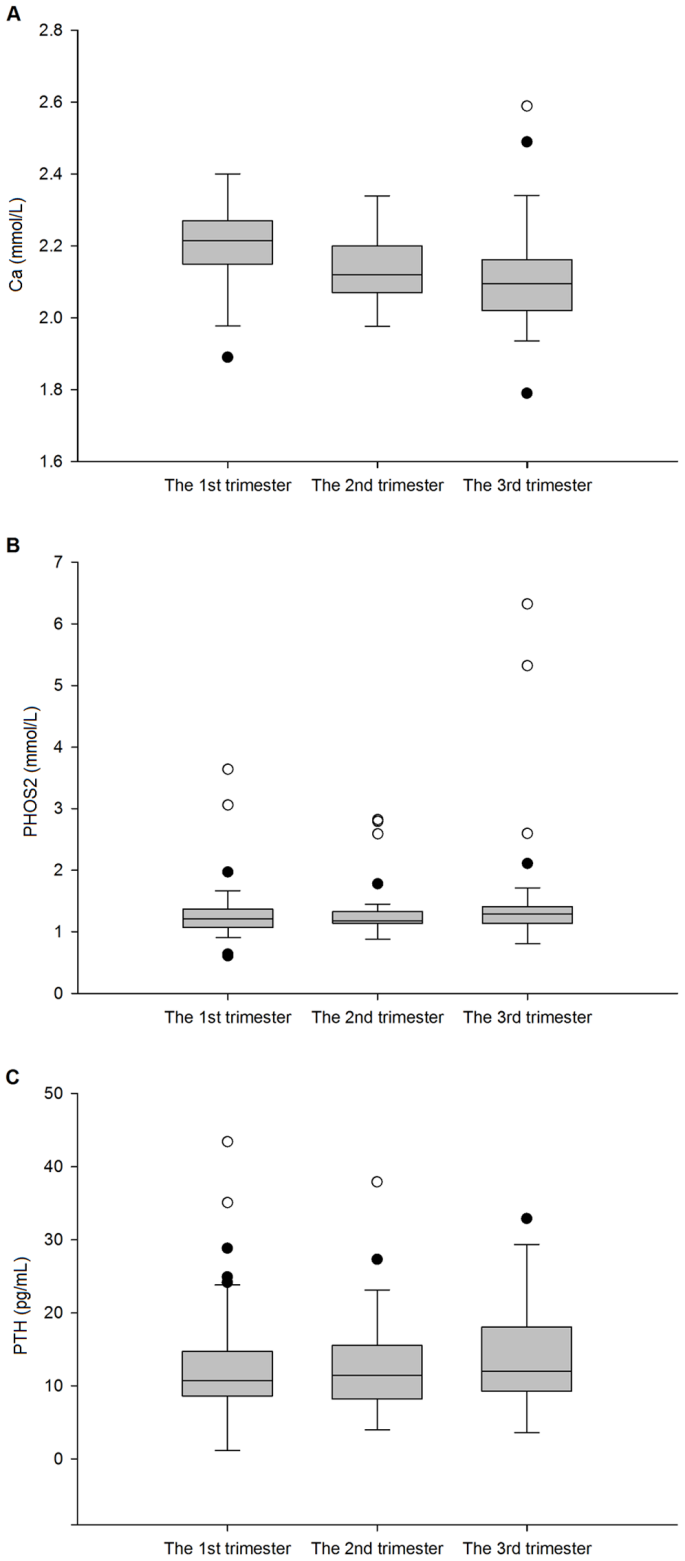
Changes in serum Ca, PHOS2 and PTH during pregnancy (n = 50). Data are presented as boxplots. Serum Ca levels of the 2^nd^ and the 3^rd^ trimester were significantly lower than the 1^st^ trimester (p = 0.000), and with no changes during the latter two trimesters (p = 0.334) (A). No significant changes were observed in levels of PHOS2 (p = 0.288) (B) and PTH (p>0.279) (C) during pregnancy. Ca, calcium; PHOS2, phosphate; PTH, parathyroid hormone; •, outliers; ○, extreme values.

The 25(OH)D was significantly higher during the 2^nd^ and the 3^rd^ trimesters than the 1^st^ trimester (p = 0.000). However, no significant trend was seen between the last two trimesters (p = 0.416) (Figure 1). Interestingly, opposite changes were observed in Ca, which was significantly lower during the 2^nd^ and the 3^rd^ trimesters than the 1^st^ trimester (p = 0.000), and was unchanged during the last two trimesters (p = 0.334) (Figure 2A). No significant changes were observed in levels of PHOS2 (p = 0.288) (Figure 2B) and PTH (p>0.279) (Figure 2C) during pregnancy.

### Changes of 25(OH)D levels between summer and winter

After classifying each trimester into S and W subgroups, no significant difference was seen in the S/W ratio (27/23, 34/16, 23/27, respectively; p = 0.081). The median level of 25(OH)D of the S subgroup was higher than the W subgroup during the 1^st^ trimester, but only showed a trend (p = 0.061). However, if the statistical test was one-tailed, p value was significant, while the S subgroup was significantly higher than the W subgroup in the next two trimesters (p = 0.000 and p = 0.005, respectively) (Figure 1).

### Associations between 25(OH)D and Ca, PHOS2, PTH and season in each trimester

A multiple linear regression model was employed to assay associations between 25(OH)D and Ca, PHOS2, PTH, and season in each trimester. During the 1^st^ trimester, a significant positive association was found between 25(OH)D and season (B = 7.31, p = 0.005), as well as Ca (B = 38.56, p = 0.006) after adjusting for age, R^2^ = 0.36. After adjusting for age, season (B = 13.07, p = 0.001) and Ca (B = 43.21, p = 0.037) were significantly positively associated with 25(OH)D during the 2^nd^ trimester, R^2^ = 0.25. Only season (B = 12.32, p = 0.011) was significantly positively associated with 25(OH)D during the 3^rd^ trimester after adjusting for age, R^2^ = 0.17. No associations were observed between 25(OH)D and PHOS2, or PTH. However, in this case, serum Ca should be seen as the consequence, and 25(OH)D as the cause.

### Associations between 25(OH)D and thyroid parameters during pregnancy

We also analyzed TSH, FT_3_, FT_4_, TPOAb and TgAb during each trimester in a multiple linear regression model simultaneously. Only FT_3_ (B = 4.09, p = 0.024) was found to be positively associated with 25(OH)D levels during the 1^st^ trimester. However, when two subjects whose FT_3_ values were higher than the upper limit of 5.70 pmol/L were excluded, the association did not attain statistical significance (p = 0.063). No significant associations were observed between 25(OH)D and other thyroid parameters in any trimester.

## Discussion

Vitamin D deficiency/insufficiency during pregnancy can cause a series of adverse outcomes for pregnant women and their offspring. Worldwide, vitamin D deficiency/insufficiency in pregnant women ranges from 5% to 83.6% [7], [13], [14], [16], [39], [40]. In our study in which calcium- or vitamin D-containing supplementation was not used, the subjects showed a surprisingly high prevalence of vitamin D deficiency of 96%, 78% and 76%, respectively, during the 1^st^, the 2^nd^ and the 3^rd^ trimesters. These rates were higher than those in a Thai study of subjects in a region near the equator. In that study, the rate of vitamin D insufficiency (serum 25(OH)D<75 nmol/l) was 83.3%, 30.9% and 27.4%, respectively, from the 1^st^ trimester to the last trimester [14]. If our study had employed the same cut-point, the rate of vitamin D insufficiency would have been even higher. The high prevalence of vitamin D deficiency in our study was probably due to (1) the relatively high latitude (41°N); (2) different eating habits between Chinese and Western populations; (3) lack of use of calcium or vitamin D supplementation before and during pregnancy.

Our study also found that the 2^nd^ trimester 25(OH)D concentration was significantly higher than the 1^st^ trimester's, and equivalent to the 3^rd^ trimester's. This interesting finding is not completely in agreement with previous studies. In Feixiang et al.'s study [40], the mean level of 25(OH)D in the 3^rd^ trimester was significantly higher than in the 2^nd^ trimester. Thus, we believe that the higher levels of 25(OH)D during pregnancy reported in some studies were partly due to the use of supplementation during pregnancy [41]. However, in present study, the specific mechanism for the increase in 25(OH)D during the 2^nd^ trimester was unclear. On the contrary, we expected that it would decrease as the gestational age increased. Generally, 1,25(OH)_2_D and not 25(OH)D levels increase, being two-fold higher in women in the 3^rd^ trimester of pregnancy than in non-pregnant or postpartum women [42], [43], since the synthesis in the kidney of 1,25(OH)_2_D increases decidual and placental CYP27B1 enzyme activity [44], and specific methylation of the placental CYP24A1 represses transcription of this gene [45]. However, our subjects did not use vitamin D or calcium supplementation before or during pregnancy, so what caused the increase of 25(OH)D levels in the 2^nd^ trimester? We attempted to interpret this perplexing finding through the perspective of gestational weight gain (GWG). Mean total GWG of normal weight adult women giving birth to full term infants ranges from a low of 10.0 to a high of 16.7 kg [46]. Reported mean rates of GWG are 0.169 kg per week in the 1^st^ trimester, 0.563 kg per week in the 2^nd^ trimester, and 0.518 kg per week during the 3^rd^ trimester [47], [48]. Although dietary intake only accounts for 5% of vitamin D sources, this evidence may explain, in part, the increased levels of 25(OH)D in the 2^nd^ trimester, and the lack of further changes in the 3^rd^ trimester. The elevated 25(OH)D concentration in the 2^nd^ trimester might be due to increased food intake causing the rate of GWG to increase sharply between the 1^st^ and the 2^nd^ trimester. However, these rates were similar between the last two trimesters, which might indicate a steady food intake; thus no changes were observed in 25(OH)D concentrations.

Among numerous factors influencing serum 25(OH)D, one that has commonly been reported is the season. Our study showed that during the 1^st^ trimester, the median level of 25(OH)D in summer was higher than in winter, but was only a trend statistically. However, if the significance level was one-tailed, p value became significant. The 25(OH)D levels in summer were significantly higher than in winter during the next two trimesters. In a multiple linear regression model, a positive relationship was found between 25(OH)D levels and seasons. These findings were in agreement with previous studies [7], [40], [49]. The abundant sunlight during summer months means enhanced levels of 25(OH)D via cutaneous synthesis. Moreover, compared to the freezing winter in Shenyang, the pleasant summer climate may encourage increased opportunities for outdoor activities.

Total serum calcium concentration falls during pregnancy, with a slight increase toward the end of gestation. This pattern parallels the alterations in serum albumin concentration caused by the increased intravascular fluid volume of pregnancy and the resulting hemodilution [50]–[52]. These changes may weaken the association between 25(OH)D and calcium, especially in the 3^rd^ trimester according to our present study. Consistent with previous studies [53], [54], our study has shown no changes in phosphate metabolism. To the best of our knowledge, no previous data that showed an association between 25(OH)D (not 1,25(OH)_2_D) and serum phosphate. Interestingly, PTH, which is usually considered the stimulus for increased renal hydroxylation of 25(OH)D to 1,25(OH)_2_D has not been shown to be increased during pregnancy [55]–[57]. Our study confirmed these findings.

As mentioned previously, we analyzed the relationship between serum 25(OH)D and thyroid parameters using a multiple linear regression model. Our study only observed a positive relationship between serum 25(OH)D and FT_3_ in the 1^st^ trimester. When two abnormal FT_3_ values were excluded, this significant relationship was attenuated to a trend. Active calcitriol regulates translocation of T_3_, at least in the cerebellum, by increasing the binding capacity of the cytosolic T_3_-binding protein [58]. At the pituitary level, vitamin D regulates TSH secretion of thyrotrophs [59]. Studies *in vitro* and *in vivo* suggest that there is an increase in the response to 1,25(OH)_2_D in the presence of T_3_ in cultured anterior pituitary cells, and that after acute administration of 1,25(OH)_2_D the TSH level increased [60]. Thus, it is understandable that once TSH levels increased, thyroid hormones would follow suit. However, others have shown that serum concentrations of TSH and the response of TSH to thyroid-releasing hormone (TRH) was lower in patients or experimental animals with high plasma concentrations of active vitamin D [61], Chailurkit et al. pointed out that high vitamin D status was associated with low circulating TSH in younger individuals [62]. Glinoer showed that the sharp increase of human chorionic gonadotropin in the 1^st^ trimester enhanced thyroid hormones and induced partial suppression of serum TSH [63], which makes this issue a bit more complicated. Together, the overall effects were that vitamin D might not change the TSH value, although the possibility that active vitamin D controlled the action of T_3_ in its negative feedback regulation could not be excluded [58]. The biological effects and mechanisms affecting the relationship between 25(OH)D and FT_3_ are unclear, and a large study sample is needed to confirm this relationship. In addition, it was reported that serum 25(OH)D values showed only a weak inverse correlation with TPOAb titers [64]. As pregnancy is a specific course which reflects the state of immune tolerance, the anti-thyroid autoantibody titers decrease by at least 50% from the 1^st^ to the 3^rd^ trimester [65]. This may have weakened the association between 25(OH)D and thyroid antibodies in present study, and therefore we did not observe any relationship. We speculate that although vitamin D deficiency and thyroid dysfunction/thyroid autoimmunity during pregnancy can contribute to some of the same adverse events, these two factors may act independently.

It is time to raise public health awareness that use of vitamin D supplementation before and during pregnancy is extremely important. According to the National Health and Nutrition Evaluation Survey, in the United States, the average dietary intake of vitamin D (including supplements) maybe as low as 200 IU per day (with differences according to age) [66]. Since there are differences between Chinese and Western eating habits, average dietary intake of vitamin D by Chinese might be lower than that level. Surprisingly, the recommended daily dose of vitamin D varies hugely from 200 IU to 2000 IU per day [38], [67]–[69]. It has been reported that for pregnant women residing in Thailand, Southeast Asia (14°N), vitamin D supplementation at 400 IU per day is likely to prevent vitamin D deficiency, but is inadequate to prevent vitamin D insufficiency even at a dose of 800 IU per day [14]. To determine the optimal supplementation dosage for Chinese, a large clinical trial needed.

The strengths of present study are that all blood samples were collected from the same subjects and measured together. Thus, we were able to explore the changes of serum 25(OH)D during pregnancy more accurately. Also, the subjects declared that they did not use vitamin D or calcium supplementation before or during pregnancy, which helped us to determine the effects of season and pregnancy *per se* on changes in vitamin D. Because of the retrospective design of the study, some limitations could not be avoided. One limitation was the sample size; 50 women seem inadequate and we lack non-pregnant samples as controls. A questionnaire was not available, thus some important data were missing, such as BMI; eating habits; use of cigarettes, alcohol, and sunscreen; outdoor activities; parity; and education, which in led to relatively low R^2^ values in the multiple linear regression model.

## Conclusions

We observed a high prevalence of vitamin D deficiency during pregnancy in women from Northeast China who did not use supplementation. Supplementation was advocated before and during pregnancy, and we speculate that the daily dose of vitamin D supplementation exceeded 800 IU per day. No significant relationships were observed between 25(OH)D concentrations and thyroid parameters during pregnancy. It is suggested that these two factors may act independently to cause some of the same adverse events seen in pregnant women and their neonates.
